# Colorimetric and electrochemical detection of pathogens in water using silver ions as a unique probe

**DOI:** 10.1038/s41598-020-68803-8

**Published:** 2020-07-20

**Authors:** Virendra Kumar, Adity Chopra, Bhawana Bisht, Vijayender Bhalla

**Affiliations:** CSIR-Institute of Microbial Technology, Sector 39A, Chandigarh, 160036 India

**Keywords:** Biological techniques, Nanoscience and technology

## Abstract

The manuscript highlights the efficacy of silver ions to act as a unique probe for the detection of bacterial contamination in water samples. The bacterial cell membrane adherence property of the silver ions was employed to develop two different bacterial detection assays employing colorimetric and electrochemical techniques. In one of the schemes, silver ion was used directly as a detector of bacteria in a colorimetric assay format, and in the other scheme surface-functionalized antibodies were used as a primary capture for specific detection of *Salmonella enterica* serovar Typhi. The colorimetric detection is based on silver-induced inhibition of urease activity and silver ion utilization by bacteria for the rapid screening of enteric pathogens in water. The specific detection of bacteria uses an antibody-based electrochemical method that employs silver as an electrochemical probe. The ability of silver to act as an electrochemical probe was investigated by employing Anodic Stripping Voltammetry (ASV) for targeted detection of *Salmonella* Typhi*.* For further insights into the developed assays, inductively coupled plasma mass spectrometry (ICP-MS) and transmission electron microscopy (TEM) studies were performed. The sensitivity of the developed assay was found to be 100 cfu mL^−1^ for colorimetric and 10 cfu mL^−1^ for electrochemical assay respectively.

## Introduction

Poor sanitation and contaminated water results in 1.7 million deaths annually in the world^1^. Common contaminants of water include enteric bacteria such as *E. coli, S. *Typhi and other non-typhoidal strains of *Salmonella* spp., *Shigella* spp. and *V*. *cholera*. The well known bacterial detection techniques include culture-based plating method^2^, quantitative real-time PCR, loop-mediated isothermal amplification^3^, ELISA^4^, paper-based microfluidics^5^, mass spectrometry, luminescence assays^6,7^ and molecular imprinting technology^8^. Gao et al. described the detection and identification of single bacterium using electrochemical collision technique^9^. But all these above techniques are expensive, time-consuming, and require expert handling. Rapid colorimetric detection methods are emerging as sensing platforms to overcome drawbacks of current existing techniques. Many research groups worldwide have used colloidal assays, employing nanomaterial-based detection^10^. There are several studies wherein receptor coated metal nanoparticles have been used for bacterial detection by utilizing unique chemical and physical properties of nanomaterials^11,12^. A colorimetric method for the detection of* E*. *coli* was developed by Raj et al. that employs AuNPs modified with cysteine as detector probes^13^. The detection sensitivity for the method was shown to be 100 cfu mL^−1^. Similarly, Wang et al. have earlier reported a method using Au coated magnetic nanoparticles conjugated with *Staphylococcus aureus* specific antibody and Surface-enhanced Raman scattering (SERS) based platform^14,15^. Another group, Zhou et al. have utilized SERS based approach employing silver nanoparticles for the detection of both Gram-positive as well as Gram-negative bacteria^16^. Abbaspour et al. made an aptamer-based sandwich assay for detection of *S. aureus* wherein silver nanoparticle conjugated aptamer was employed for development of ASV based electrochemical assay^17^. All of the above studies were based on utilizing either the electrochemical or optical properties of nanoparticles. However, nanoparticles are prone to agglomeration and require some kind of receptor layer or polymer coatings for recognition purposes. These nanoparticles based probes are not stable in the field setup and cannot stand long term storage and therefore are prone to detection inefficiencies. The synthesis of nano-bioconjugates is also time-consuming and costly because of the requirement of linkers and bioreagents^18^.

The bactericidal properties of nanoparticles have been very well demonstrated. Many studies have also attributed this action to the released metal ions^19,20^. Silver ions have shown high bactericidal activity by inhibiting DNA and protein synthesis^21^. The antibacterial property of silver ions has already been utilized in topical antiseptics and in coatings for orthopedic implants^22–24^. The silver leads to changes in protein structure and inactivation of many enzymes^25^. Silver ion disrupts the respiratory system of bacteria by inhibiting important respiratory chain proteins of cytochrome family. It induces lipid peroxidation and damages cell membranes to kill bacteria. Other than this, the mechanism of the antimicrobial action of silver ions is also known to be related to its interaction with thiol groups^26,27^, although they can also interact with other sites^28^. Urease (a nickel-based metalloenzyme) hydrolyzes urea into ammonia and carbon dioxide, the released ammonia raises the pH of the system. Silver is known to interact with urease and inhibit its urea hydrolyzing activity^29^. This interaction of silver ions with bacterial cells and urease can be exploited to develop a colorimetric assay for bacterial load estimation in water samples. Previously, silver ions have also been used for the enhanced detection of norovirus although the exact mechanism is not known in that case^30^.

**Scheme 1:**
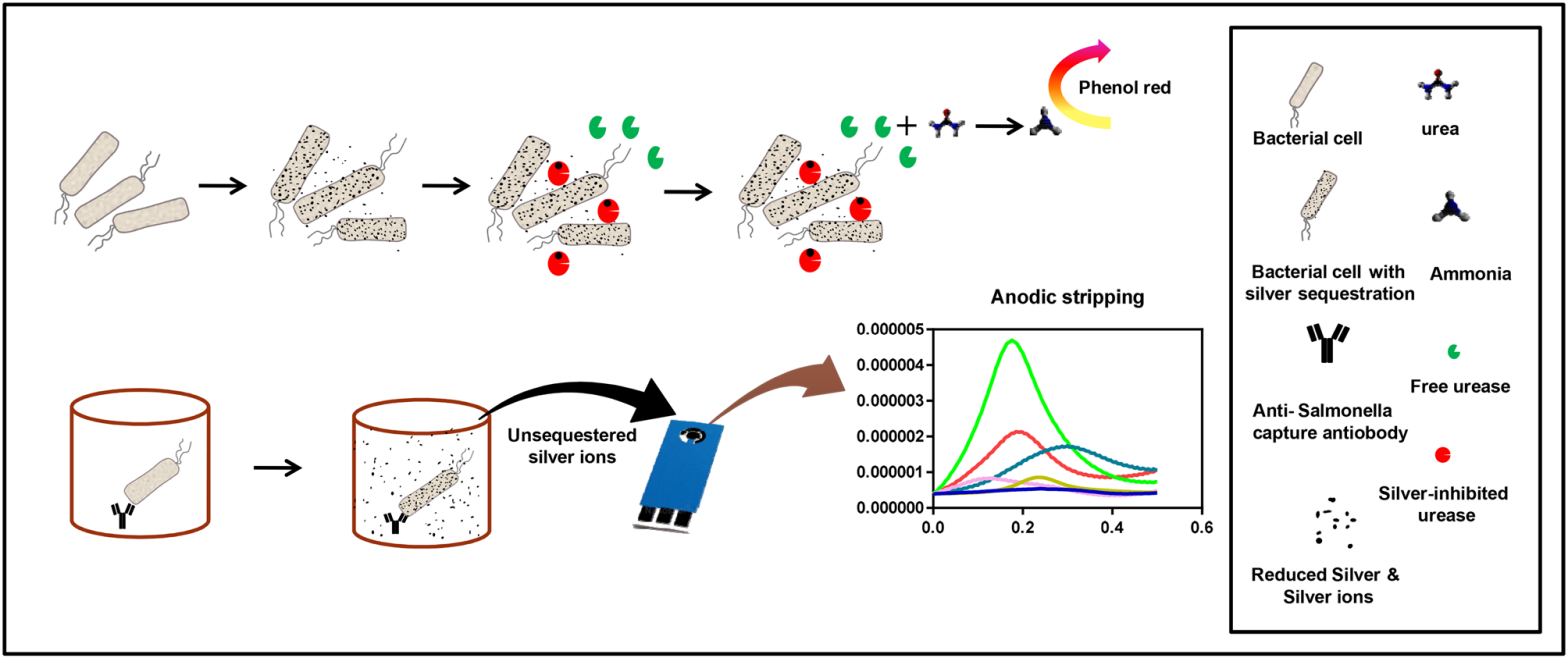
The schematic shows the two developed assay formats. The first one is a colorimetric assay based on silver sequestration phenomena in presence of bacterial cells. The introduction of the urease leads to enzymatic inhibition depending on the concentration of free silver in solution. The urea and phenol red can be conveniently used to detect enzyme activity which is directly related to the concentration of bacterial cells in sample. The second assay employs surface-functionalized antibodies and after incubation with the sample followed by silver ion. The leftover silver on top of the cells bound to the antibody layer is used as an electrochemical probe to detect presence of bacteria. Microsoft Office PowerPoint software version 2007 was used to create this image. https://products.office.com/en-in/home.

We have developed a colorimetric assay that utilizes the efficacy of silver ion to interact with bacterial cell membrane and employs the catalytic activity of urease for the development of a rapid assay for detection of bacteria in contaminated water. This particular scheme obviates the need for having a bioreceptor to detect the total coliform bacteria which is matter of big concern for public health departments worldover. Basically, when a defined concentration of silver ions is introduced into contaminated water, silver ions get sequestered by enteric pathogens in a concentration-dependent manner. The amount of leftover silver ions after sequestration inhibits the catalytic activity of urease in a concentration dependent manner. Thus, by correlating to a convenient enzyme assay one could define bacterial concentration in water which is the main theme of the present paper. The urease activity can be conveniently monitored through naked eye by observing changes in the pH upon the addition of urea and phenol red indicator. This one, makes a simple sensor for detection of coliform bacteria in water. Nevertheless, for specific detection of particular kind of enteric strains, we have described another approach that employs antibody as a primary capture and silver ions as a detector probe in electrochemical assay. Scheme 1 illustrates the sequential graphical representation of the reactions and developed assay platforms as described in this paper.

## Results

### Silver ion as a receptor probe for detection of bacteria

**Figure 1:**
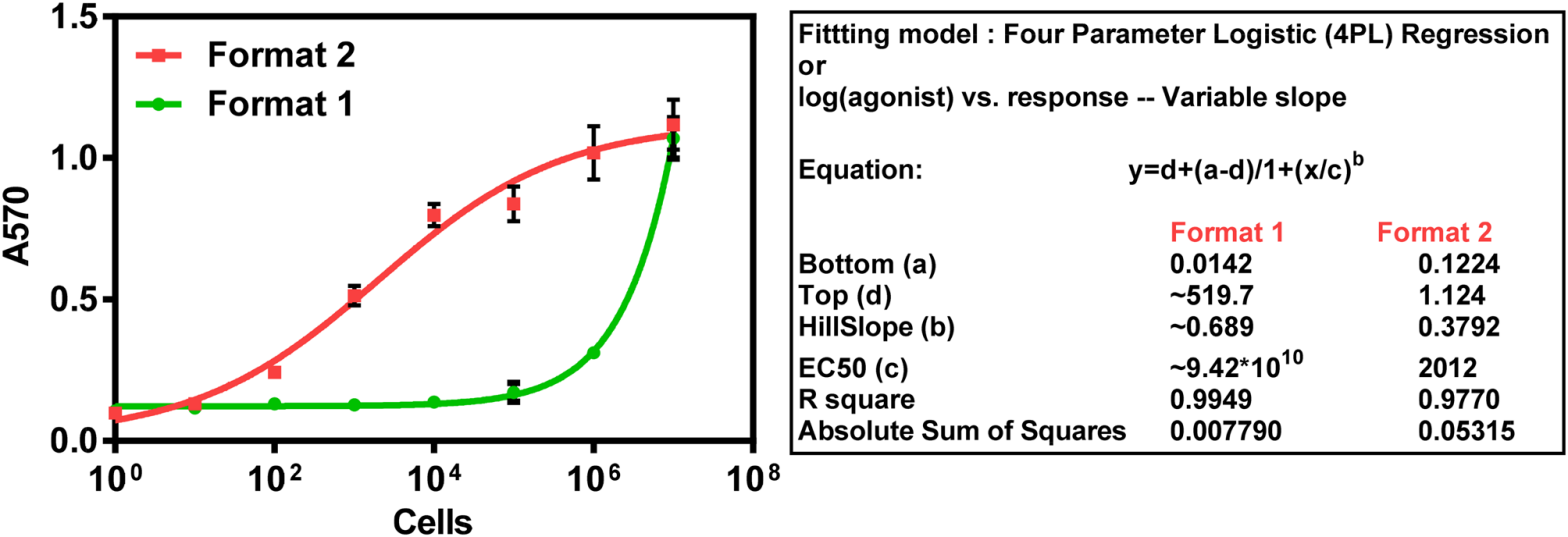
Inhibition of urease activity by silver ions in presence of serial cell dilutions. In format 1, bacterial cells were introduced after urease and silver ions. In format 2, cells were incubated initially with silver ions before adding urease.

To elucidate the urease inhibition property of silver in a colorimetric assay format, silver nitrate dilutions were tested in nM range. Figure S1 shows that significant inhibition of 20 µg urease (4 µL of 5 mg mL^−1^) by silver ions occurs at a working concentration of 5 nM in a total reaction volume of 1 mL. Thus anything above that concentration could be taken to perform the assay in the presence of bacteria and it is a matter of optimization to find out particular concentration that gives perfect analytical signal to cover the entire range of tested concentrations. An experimental graph (Fig S2) in supplementary information shows the performance of different working concentrations of silver ions (5–200 nM) in the standard assay. The Fig S2 shows best quantitative response at 100 nM working concentration of silver. In the reaction, 400 µL cell suspension was treated with 400 µL of silver nitrate (250 nM stock, working concentration is 100 nM), and to this 20 µg urease was added and mixed with 200 µL colorimetric substrate (30 mM urea in 66.67 µg mL^−1^ phenol red). After an incubation period of 10 min absorbance was measured at 570 nm. Two formats of the assay, with differences in sequence of addition of reagents were performed as described under methods; in both the formats an increase in enzyme activity was observed with increase in the concentration of bacterial cells due to sequestration of silver ions by bacteria as seen in Fig. 1. The resultant enzyme activity was quantified by addition of urea substrate along with pH indicating dye (phenol red). Samples with high cell count show lesser urease inhibition, thus more O.D of pink colour was observed whereas samples with less or no cells showed urease inhibition effect (yellow colour). As seen in Fig. 1, a linear decrease in urease activity was observed in the second assay (format 2) in marked contrast to the assay format 1 wherein the amount of silver ions is not correlating to bacterial load in the sample as the urease gets inhibited during the initial interaction with silver. Thus sequence of addition of reagent is very important and format 2 is the main suggested method and was also followed for further assays with real water sample analysis.

### TEM analysis for silver sequestration by bacterial cells

**Figure 2:**
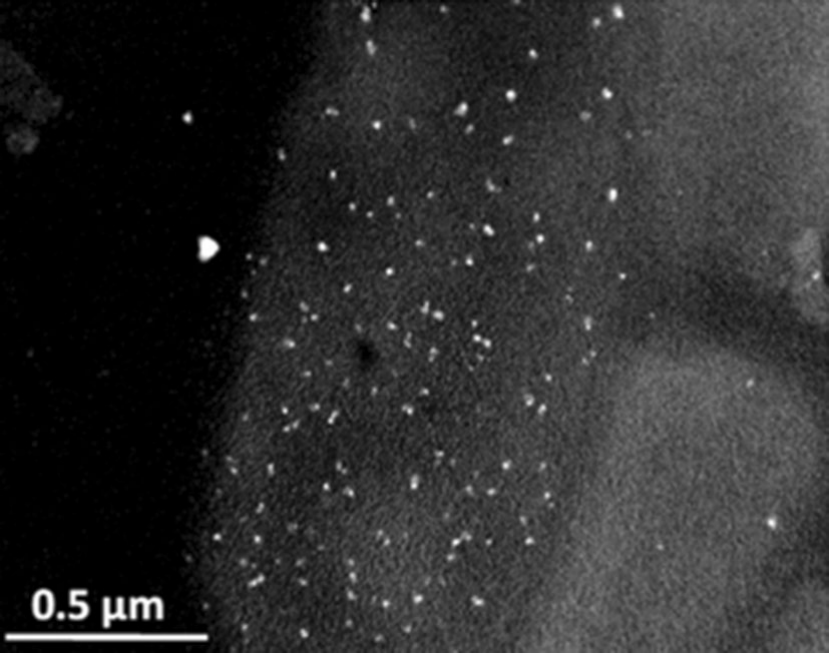
TEM image shows the reduced silver ions inside and on bacterial cells wall.

To visualize the silver sequestration phenomenon the unstained bacterial cells were observed under TEM for the presence of dense silver particles. Bacterial treatment with silver ions is known to accumulate silver particles both outside and inside the cell due to the presence of reducing enzymes and reducing sugar moieties on the cell surface that reduces the metal cation^19,21^. Figure 2 is a TEM image that shows the ability of bacterial cells to take up silver ions and reduce them into small metallic particles inside and on the surface of bacterial cells. Silver ions mixed with different bacterial dilutions got distributed immediately inside and on the surface of bacterial cells after an incubation of 10 min. According to literature, silver ions enter inside both Gram-positive as well as Gram-negative bacteria but their route of entry is different. The silver ions initially bind with the outer cell membrane through glutamic acid and teichoic acid on the cell surface and later enters the cell through porin proteins in case of Gram-negative bacteria within 30 min leading to leakage of cytoplasmic contents^27,31^. Therefore in the present study, the bacterial cells were exposed for 10 min with silver ions in order to get maximum sequestration with negligible cell lysis. Thus our duration of cell incubation with silver ions supports the previous reports that have shown cell breakage and release of cytoplasmic contents from bacterial cells on treatment with silver ions for a period of 1 hour^27^. Although cell lysis will not affect assay results drastically, because of cellular content after cell lysis will not release many silver ions. Silver ion will stay reduced with cellular components with very less oxidation outside the cells. Still, cells were treated with the silver ion solution for short durations only, we can see intact bacterial cells in TEM image and no lysis was observed.

### Quantification of silver sequestration using inductively coupled plasma mass spectrometry (ICP-MS)

**Figure 3:**
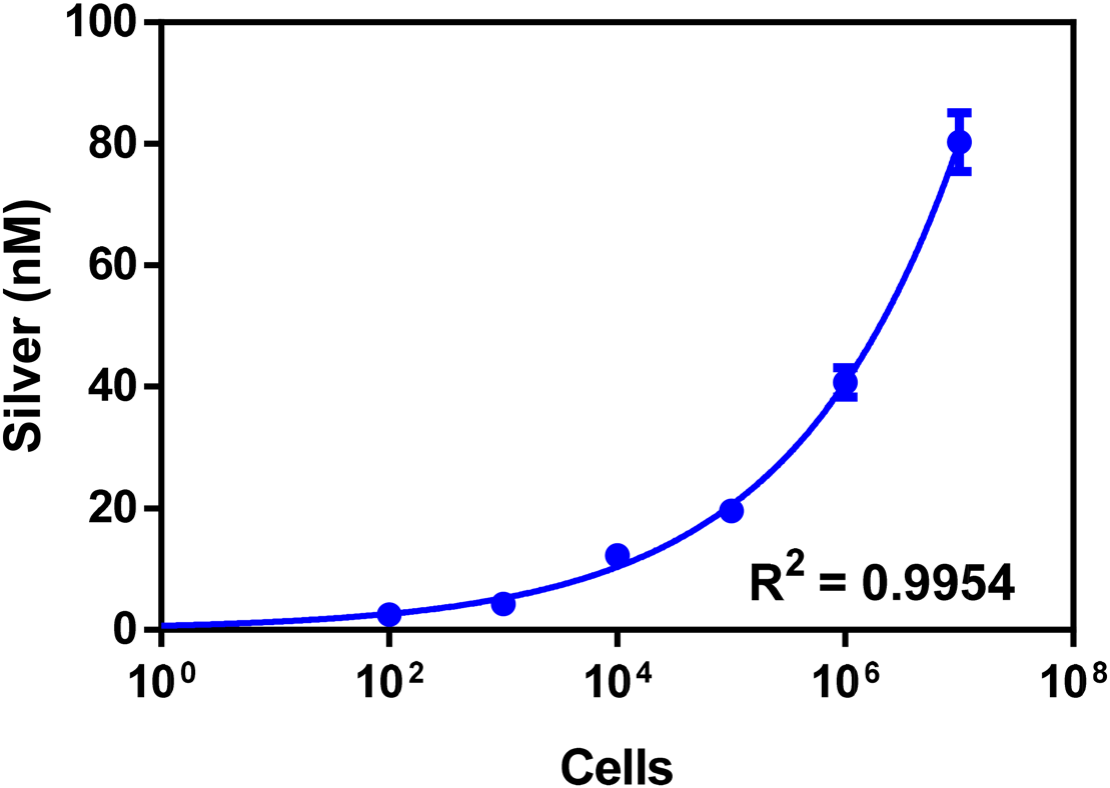
Quantification of silver ion sequestered by bacterial cells. Serially diluted cells were treated with 100 nM silver ions and after washing unsequestered silver ions, ICP-MS is performed on centrifuged cells to quantify sequestered silver ions.

ICP-MS is a very sensitive mass spectrometer that ionizes the sample using inductively coupled plasma and it creates atomic ions to detect the metallic ions in the sample. It exhibits the limit of detection in the parts-per-trillion range^32^. Elemental analysis through ICP-MS involves sample digestion and disruption of the sample matrix to form a homogenous solution that can be passed through the nebulizer. These characteristics make ICP-MS compatible for the quantification of silver inside the microbial cells. Till now there are few studies that used elemental quantification with bacterial samples. Lin and Hamme have used antibody labeled gold nanoparticles for the detection of Salmonella and *E*. *coli* through ICP-MS that gives a quantitative detection of the pathogens in the contaminated samples by measuring the number of labeled gold nanostructures bound to the pathogens^33^. Li et al. detect bacterial load by using specific antibody-gold nanoparticle conjugate, and digesting pathogen-bound gold nanoparticle conjugate and analyzing it with ICP-MS of gold ions^34^. Figure 3 shows the intake of silver ions by the bacterial cell dilutions (0–10^7^ cfu mL^−1^), the maximum concentration of sequestered silver ions was observed at 10^7^ cfu mL^−1^. ~ 82% sequestration was observed with 10^7^ cfu mL^−1^ at 100 nM silver ion treatment. Figure 3 shows the sequestered silver is directly proportional to the concentration of the cells.

### Detection of enteric bacteria in real water samples

**Figure 4:**
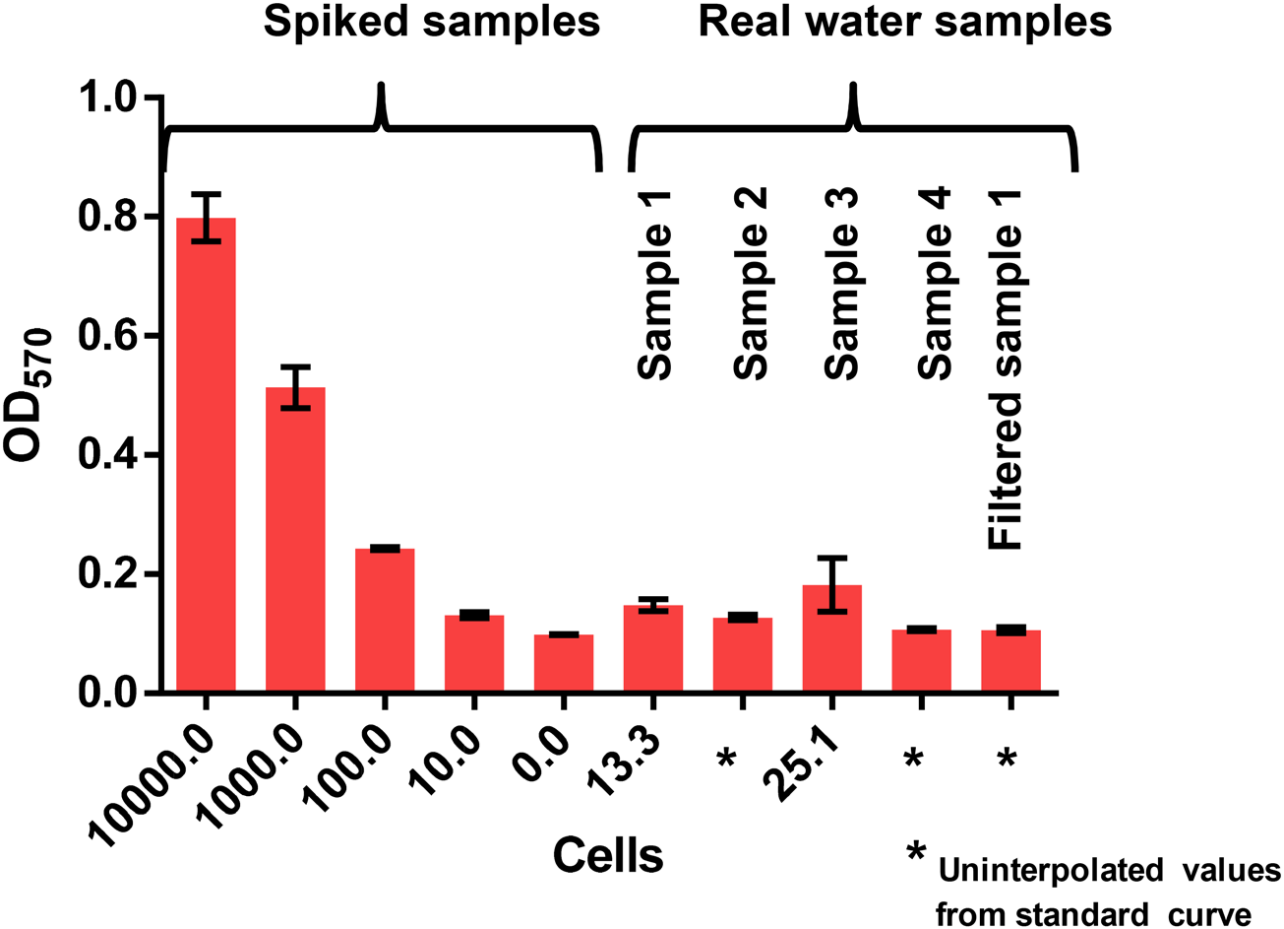
Optical detection of bacterial load in water samples from different sites, compared with spiked samples. Spiked water samples are the part of the standard curve (Fig S3) from which the unknown bacterial load (cfu mL^−1^) were interpolated.

The developed optical assay was used for the detection of microbial load in real water samples. Standard curve and sample preparation was performed as explained in results and discussion. Different samples of tap water were taken from four different sites. Figure 4 represents the curves with bacterial spiked samples and real water samples. For standard curve preparation (Fig S3), salmonella was used as a model organism to represent enteric bacteria in bacterial spiked samples. Water samples 1, 2, and 4 show comparatively less urease activity that suggests low or no bacterial load in the water samples. However, in the case of sample 3, a higher urease activity was seen suggesting a high bacterial load. In sample 3 a maximum load of ~ 25 cfu mL^−1^ was observed. To check the interference by other components of water other than bacteria, sample 1 was filtered through 0.22 micron filter and tested, but no significant interference was observed in the filtered sample 1 as seen in Fig. 4. The recovery percentage and the assay repeatability were also checked (data is shown in supplementary, Fig S8). Recovery percentage is a feature of cell concentration but at 10^3^ cfu mL^−1^ 98.2 ± 9% recovery was found. Therefore the developed assay could be used as a method for detection of enteric bacterial load in drinking water samples.

### Specific detection using antibody and silver ions as an electrochemical probe

**Figure 5:**
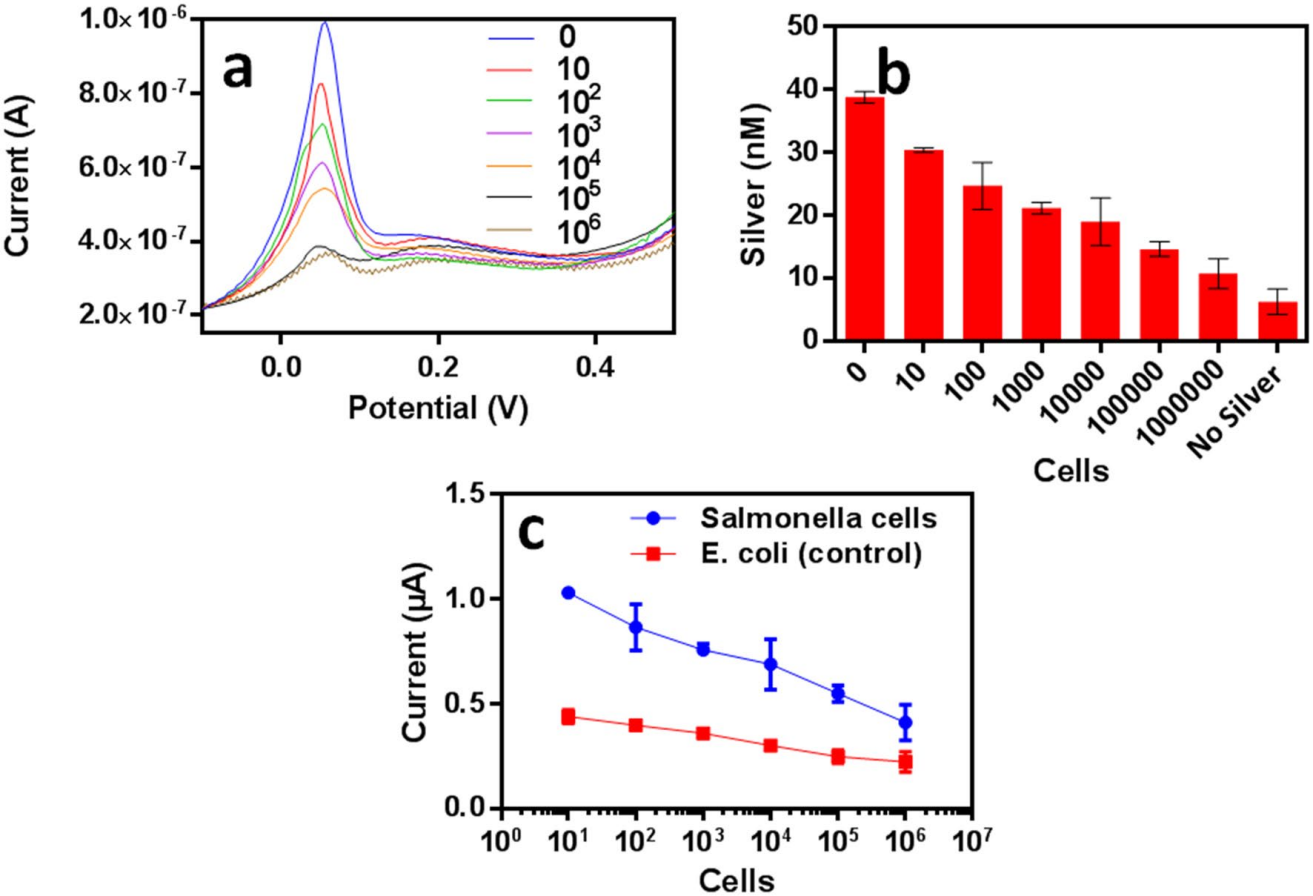
Electrochemical detection of *S.* Typhi using anti-salmonella antibody and silver ions as a probe (**a**) square wave voltammograms using anodic stripping voltammetry for unsequestered silver ions at different cell dilutions, shows a decrease in peak current with increase in cell dilutions. (**b**) The Unsequested silver ion concentration for different cell dilutions. (**c**) The specificity of assay towards *S.* Typhi when compared with *E. coli*.

Among the enteric bacteria, *S.* Typhi is one of the most significant causes of morbidity; therefore we have focused our attention on developing a specific electrochemical detection technique to detect salmonella in water samples. The assay makes use of our in house generated anti-salmonella antibody as a capture molecule (Scheme 1). The silver ions sequestered by cells (captured by anti-salmonella antibody) are difficult to detect since these silver ions get reduced to form small silver particulates. In order to estimate sequestered silver ions, oxidation of these silver ions is required but, the cell lysis hinders the procedure of oxidation of reduced silver ions. Therefore in our scheme, we have used the leftover unsequestered portion of silver ions for the ASV mediated electrochemical detection. Previously also there are some reports that describe the use of ASV in the quantification of silver metal ions released from catheters and their uptake by bacterial biofilms by exposing the silver-coated catheters to the synthetic urine samples^35^. The most important part of our developed assay is the choice of blocker that should effectively repel Ag^+^ ions to settle onto the sensing surface. We observed that 2% PVP acts as a very effective non-protein blocking agent. Figure 5a shows square wave voltammograms of unsequestered silver ions at different cell dilutions (0–10^6^). The voltammograms show a concentration-dependent decrease in the current with increase in cell concentrations. A standard curve was used to determine the concentration of silver ions in the sample for a range of 0–500 nM of silver (Fig. S4). Figure 5b shows unsequestered silver ion concentration in the supernatants of different cell dilutions. Figure 5c shows the specificity of the developed electrochemical assay towards the *S.* Typhi in marked comparison to *E. coli*. The sensitivity of the assay was found to be 10 cfu mL^−1^ and as seen in table ST9 in supplementary data, the developed assay shows better sensitivity and requires less detection time in comparison to other available assays. Before developing an antibody based specific electrochemical assay, a non-specific detection of bacterial cells (without antibody) using Anodic stripping voltammetry on screen-printed carbon electrodes (SPCE), was also performed by quantitation of unsequestered silver ions (Fig S5). However this has its inherent limitations and antibody based method is the main suggested method for specific detection.

### Interference study of heavy metal ions, and other inorganic contaminants on urease inhibition assay

**Figure 6:**
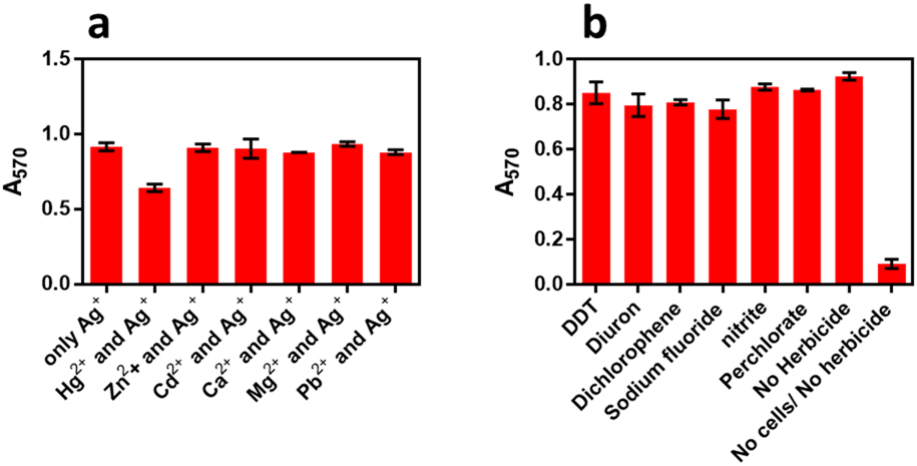
(**a**) Different metal ions tested for interference to the optical assay. In clean water 100 nM heavy metal ions were tested in presence of 100 nM silver ions and fixed 10^5^ cfu mL^−1^ concentration of bacterial cells. (**b**) The Interference due to some herbicide and chemical contaminants was tested at a concentration of 1 µg mL^−1^ with our routine optimized assay at 100 nM silver ions concentration and 10^5^ cfu mL^−1^ of cell dilution.

The effect of different metal ions was checked for interference to the urease inhibition assay. Inhibition by metal ions on the enzyme is highly dependent on the nature of the ion, accessibility of the active site and amino acids involved in the active site of the enzyme^36^. It is very important to check the effect of other metal ions since some ions can act allosterically and can enhance the enzymatic activity while others may reduce the enzymatic activity by changing its conformation upon binding^37,38^. Figure 6a shows the result of interference due to different metal ions. The presence of Hg^2+^ ions in the standard reaction mixture can significantly perturb the optical signal (Fig S6). No significant interference in optical assay was observed due to Mg^2+^, Cd^2+^, Ca^2+^ Pb^2+^, and Zn^2+^ ions. The working concentration of all the metal ions was 100 nM. The effect on urease inhibition, by Hg^2+^ ion was due to its binding to the thiols of the enzyme similar to the effect with silver ions, however, silver ions also bind to other N- and O-containing groups^39^. Mercury is previously known to inhibit urease noncompetitively and irreversibly^40^. Figure 6b shows interference study carried out with other water-based contaminants such as herbicides, nitrite, perchlorate, and fluoride. No significant interference was observed with any of the common contaminants. This is an along expected lines, as these compounds cause no inhibition to the urease activity even at a very high concentration as explained by Wimaladasa and Wickramasinghe^41^. The interference due to some life element ions was also checked and there was no significant interference observed by these metal ions, except Cu^2+^ ion which is previously also known to be a mild inhibitor of urease^36^ (Fig S7).

## Discussion

An assay system has been developed that could be used as a cost-effective tool for the detection of bacterial load in drinking water and employs silver as a probe for detection. In the colorimetric format, we could detect coliform bacteria varying from a very low 100 cfu mL^−1^ count to a considerably higher cell concentration up to 10^7^ cfu mL^−1^. For the specific detection of particular kinds of bacteria we have described a electrochemical format of the assay that can be used for the specific detection of *S.* Typhi in the drinking water samples. The study also sheds light on the silver sequestration phenomenon by the bacterial cells. Silver sequestration depends on the number of bacterial cells present in the solution and exposure time of silver ions with these cells. Around 82% of sequestration was observed at 100 nM silver ions concentration in the presence of 10^7^ cells. The developed system has the potential for future applications and may act as a rapid screening tool for testing of pathogens in water.

## Material and methods

### Materials

Jack bean urease and silver nitrate were purchased from Sigma-Aldrich. Urease was purchased from Sisco Research Laboratory Pvt. Ltd. Phenol Red (pH indicator dye) by Merck India Ltd. Screen-printed electrodes (TE 100) were procured from Zensor Taiwan. All the chemicals used in this study were of reagent grade and used as such without any further purification. The bacterial culture of *Salmonella* Typhi was available in our lab. *S*. Typhi was streaked on a nutrient media plate; kept overnight and fresh cells were harvested from the plate for further culturing. The cells were grown at 37 °C till 1 OD_600_ and serial dilutions in double distilled water (DDH_2_O) were made from the cell suspension. GraphPad Prism software version 6.01 (URL: https://www.graphpad.com/scientific-software/prism/) was used to make graphical images and statistical analysis. Microsoft Office version 2007 (https://products.office.com/en-in/home) was used for article writing and graphical editing.

### Urease inhibition assay

*S.* Typhi was taken as a model organism to show the ability of bacterial cells to sequester silver ions and spiked water samples were prepared with serially diluted cell concentrations from 10 to 10^7^ cfu mL^−1^. For performance evaluation purposes, the assay was performed in two different formats. In the first format urease and silver ions were incubated first and then cells were introduced. In the second format, urease was added after incubation of cells with silver ions. Thereafter urease, urea substrate, and phenol red were added sequentially to different bacterial dilutions and the absorbance of the was taken at 570 nm.

### Detection of enteric pathogens in real water samples

To analyze the cell concentrations in water samples using the developed optical assay. Tap water samples were taken from IMTECH, and from other sites in Chandigarh. These water samples (10 mL) were centrifuged and reconstituted to 400 µL. The pH of the samples was adjusted to match the distilled water. Further optimized concentration of silver ions, urease, urea-phenol red substrate were added sequentially to different samples. The absorbance was recorded at 570 nm. The concentration of cells was interpolated through a standard curve prepared using a known number of bacterial cells. To check the interference in the assay due to other components except bacterial cells, the contaminated water sample was passed through 0.22 µm filter to remove bacterial cells, and then this filtered water was checked on colorimetric assay.

### TEM analysis

TEM analysis was carried out using a Jeol Jem 2100 microscope operating at an accelerating voltage of 200 kV. Bacterial cells at 10^6^ cfu mL^−1^ were incubated for 10 min at room temperature with a silver ions at a concentration of 100 nM in DDH_2_O. The mixture was then centrifuged at 4,000 rpm for 20 min to obtain the cell pellet and then suspended in 1 mL DDH_2_O after wash step . TEM Specimen was prepared by placing one or two drops of sample on a carbon-coated-copper TEM grid (300 mesh size), and letting it dry under ambient conditions.

### ICP-MS studies of silver sequestration by bacteria

ICP-MS Thermo Electron model X7 Series equipped with PlasmaLab Software was used at Punjab Biotechnology incubator, Mohali Punjab. The instrument was operated in a Class-100 clean, laboratory at a temperature of 20 °C. Bacterial cells were diluted from 10^7^ to 0 cfu mL^−1^ with a silver ion working concentration of 100 nM in DDH_2_O. The mixture was incubated for 10 min at room temperature and then centrifuged at 4,000 rpm for 20 min to obtain the cell pellet and then diluted with DDH_2_O. For the specimen preparation, 1 mL of the sample was digested using 1 mL of HNO_3_ and the mixture was reconstituted up to 10 mL in a test tube which was later injected through the nebulizer into the instrument. The concentration of silver ions inside the cells was measured using controls.

### Electrochemical detection of *S.* Typhi

A polyclonal antibody against *S.* Typhi was used as a capture molecule for the bacterial immobilization on the micro-titer plate. The anti-Salmonella antibody was immobilized by incubation of 100 µL of 10 µg mL^−1^ antibody in bicarbonate buffer pH 9.6 overnight at 4 °C. The reaction was blocked using 2% PVP (polyvinyl pyrrolidone). Serially diluted bacterial cells were then incubated with immobilized antibodies for one hour. After washing unbound cells, 50 nM of silver ions were added to the captured cells and incubated for 10 min. The quantitation of bacteria depends on the silver ion sequestered by the cells that was estimated by Anodic stripping voltammetry on screen-printed carbon electrodes (SPCE). Measurements were carried out in 0.1 N HNO_3_ as the supporting electrolyte medium^42^. A standard curve for silver ions in 0.1 N HNO_3_ was prepared by recording the current peaks obtained by anodic stripping using the concentrations from 400 to 0 nM. 1:1 ratio of assay solution containing different concentrations of silver ions and 0.1 N HNO_3_ was mixed to be used in the electrochemical measurements. 50 µL of the prepared samples were placed in droplet form on modified SPCE surface and potential of − 0.4 V for 150 s was applied to accumulate the analyte. Square wave voltammograms were recorded over 0.2 to 0.6 V range (with 200 mV s^−1^ scan rate, 50 mV pulse amplitude and 4 ms pulse time). All measurements were carried out at room temperature (25 °C). Samples with silver ions were reduced on the surface of the electrode by using bulk-electrolysis technique followed by square wave voltammetry to oxidize the deposited silver ions and measuring the peak current. The control reaction contained assay mixture without bacterial cells, mixed with equal concentrations of 0.1 N HNO_3_.

### Interference by heavy metal ions and various inorganic contaminants, in a urease inhibition assay

To check the effect of different heavy metal ions on urease inhibition assay, we took various metal ions such as Hg^2+^, Zn^2+^, Cd^2+^, Mg^2+^, Pb^2+^ at a concentration of 100 nM (equivalent to silver ions used) and 10^5^ cfu mL^−1^ bacterial cell dilution. A reaction mixture where only silver ion was used without any other metal ions was taken as a positive control and a cell dilution without any metal ion was taken as negative control. The urease inhibition due to heavy metal ions without cells was also conducted to find possible inhibitor of urease that can hinder the optical assay result (Fig S6). A range of inorganic contaminants such as, Diuron, sodium fluoride, nitrite, and perchlorate were also checked for interference in urease inhibition assay. Various contaminants were tested at a concentration of 1 µg mL^−1^ using a sample of cells at a dilution of 10^5^ cfu mL^−1^. Samples with no herbicide and no cells were used as controls. Furthermore, silver ions, urease, urea-phenol red substrate were added sequentially to these samples with heavy metal ions and inorganic contaminants. The absorbance was recorded at 570 nm.

## Supplementary information

Supplementary file1 (DOCX 284 kb)
