# Supplementary material for: Colorimetric and electrochemical detection of pathogens in water using silver ions as a unique probe
Source: Sci Rep. 2020 Jul 20;10:11986. doi: 10.1038/s41598-020-68803-8 (PMC7371729; doi:10.1038/s41598-020-68803-8)
Supplement: Supplementary file 1 — Supplementary file1 (DOCX 284 kb) [file 41598_2020_68803_MOESM1_ESM.docx]

**Supplementary Information**

**Colorimetric and electrochemical detection of pathogens in water using silver ions as a unique probe.**

**Virendra Kumar^1*^, Adity Chopra^1*^, Bhawana Bisht^1^, Vijayender Bhalla^#1^**

^1^ CSIR-Institute of Microbial Technology, Sector 39A Chandigarh India 160036

* Both authors contributed equally

^#^ Corresponding author

E-mail address: [**vkbhalla@imtech.res.in**](mailto:vkbhalla@imtech.res.in)

***ST1. Silver ions as an inhibitor for urease enzymatic activity***

We initially studied the urease inhibition by silver ions. Fig S1a shows urease inhibition by different concentrations of silver ions. Fig S1b represents inhibition of 20 µg (4 µL of 5 mg mL^-1^) urease by silver ion at a concentration range of 5 to 500 nM in a total reaction volume of 1 mL made by adding 200 uL urea-phenol substrate (30 mM urea in 66.67 ug/ml phenol red or 2 mg phenol red in 30 ml 30 mM urea), change in colour was monitored and absorbance at 570 nm was recorded. A change in colour intensity from pink to bright yellow indicates an increase in urease inhibition (Fig. S1b). As low as 5 nM of silver ions were able to inhibit 20 µg of urease.

Fig S1 a) Urease and silver ion concentration optimization for urease inhibition assay b) 20 µg urease inhibition at different concentrations of silver nitrate 5 to 500 nM.

***ST2 Selection of optimum silver concentration to achieve the best quantitative curve for the optical detection of bacteria***

To cover for pH variation, environmental factors and presence of cells in sample; concentration of silver ions (5 nM to 200 nM) were tested to select an optimum value at which the best quantitative standard curve could be achieved to detect the bacterial load. Since the cells sequester a significant amount of silver ions depending on their number in the reaction mixture silver ion working concentrations of 5 nM and 50 nM were not able to exert the urease inhibition effect their presence. At 100 nM concentration of silver, a good window in OD_570_ was observed with respect to different tested cell dilutions and a sigmoidal curve was obtained (Fig S2). The inhibition of urease is inversely proportional to cell concentration. The activity of urease and sequestration of silver in cells is directly proportional to the cell concentration. The data was fitted using four parameters logistic regression model (variable slope).

**y= d + (a + d)/1 + (x / c)^b^**

**x** = the independent variable

**y** = the dependent variable

**a** = the minimum value that can be obtained (i.e. what happens at 0 dose)

**d** = the maximum value that can be obtained (i.e. what happens at infinite dose)

**c** = the point of inflection (i.e. the point on the S-shaped curve halfway between **a** and **d**)

**b** = Hill’s slope of the curve (i.e. this is related to the steepness of the curve at point **c**).

Fig S2 Different concentrations of silver ions were tested to achieve the best quantitative curve. Curves are fitted using four parameter logistic regression. 20 µg urease, with different cell dilutions (0 to 10^6^ Cfu mL^-1^) at different working concentrations of silver ions (5 to 200 nM) were tested. The final reaction volume was 1004 µL made by adding 400 µL cells suspensions, 400 µL silver ions, 4 µL urease and 200 µL urea-phenol red substrate. In the squared box, fitting data for the curves obtained at different concentration of solver 100 and 200 nM were compared to show that 100 nM of silver ion shows relatively lower EC50.

***ST3 Urease inhibition standard curve for bacterial load detection in real samples***

Known concentrations of bacterial cells were treated with optimized silver ion concentration for sequestration and then treated with urease and urea-phenol red substrate. Urease activity was measured in colour change from yellow to pink by optical measurement at 570 nm. This curve was used to interpolate unknown bacterial load in contaminated water.

Fig S3 Standard curve for urease inhibition assay for interpolation of unknown bacterial load in contaminated water.

***ST4 Electrochemical estimation of silver ions sequestered by the cells***

*S*. Typhi cells at different cell dilutions were incubated with 100 nM silver ions and centrifuged. The supernatant was analyzed electrochemically for unsequestered silver ions remained in solution. A known concentration of silver ions was prepared for the standard curve to interpolate unknown concentrations of unsequestered silver ions. Fig S2 shows the current obtained with known and increasing concentration of silver, By using this standard curve, we were able to estimate the silver ions sequestered and free silver ions in solution.

Fig S4 Standard curve for current intensity at different silver ion concentrations.

**ST5 *Electrochemical detection of Silver ions in the bacterial spiked sample***

The study the sequestration phenomena in the presence of bacterial cells initial experiments were performed by incubating silver ions with bacteria without employing any antibody. The quantitation of unsequestered silver ions was carried out using Anodic stripping voltammetry on screen-printed carbon electrodes (SPCE). Fig S5a shows square wave voltammograms for silver ions at different bacterial cell concentrations depicting a linear decrease in current with increase in cell concentration. The inset to Fig S5a shows a linear decrease in current with increase in cell number. The results depict that the amount of unsequestered silver ion was high at low bacterial concentration showing a saturated current from 10^4^ to 0 cfu mL^-1^. Fig S5b shows silver ion concentration in cell dilution supernatants. By subtracting silver ion concentration in each cell dilution supernatant from the total silver ions added, sequestered silver ion concentration can be depicted. Fig S5c shows the optical and electrochemical response in one comparison graph.

Fig.S5 Electrochemical studies for the quantification of the silver ions in the assay a) Square wave voltammograms after anodic stripping for unsequestered silver ions for different cell count and the inset shows the current measured for silver ions in supernatants at different cell dilutions. b) Silver ions concentration per cell dilution supernatants. c) Electrochemical and optical response by different concentrations of cells in the assay. In optical assay absorbance at 570 nm is increasing in a concentration-dependent manner, whereas in electrochemical assay the increase in the current is inversely proportional to cell concentration as unsequestered (supernatant after sequestration) silver ions was taken.

***ST6 Urease inhibition by other heavy metal ions in comparison with the silver ions***

The highest inhibition was observed with silver ion, followed by the mercury ions. There was no significant inhibition by other metal ions (Fig. S6). The assay conditions were the same as described in the main manuscript, except there were no bacterial cells were used.

Fig S6. Inhibition of urease by other metal ions without the presence of bacterial cells.

***ST7 Interference in optical assay due to normal life metal ions***

The optical assay was tested for the interference of life metal ions like Na^+^, K^+^, Cu^2+^, Co^2+^, Fe^3+^, and Fe^2+^. 100 nM working concentration of metal ions was used and all other reagents were in the same concentration as the standard optical assay. There was no significant change due to tested life metal ions in the assay, except Cu^2+^ ion, where there is a slight decrease in the optical density was observed as Cu^2+^ is a mild inhibitor of the urease enzyme^6^ (Fig S7).

Fig S7 Interference in optical assay due to life metal ions. 100 nM life metal ions, 100 nM silver ion, 20 µg of urease, 10^5^cfu mL^-1^ cells, and 200 µL urea-phenol red substrate were used in this assay.

***ST8 Recovery percentage and repeatability of the assay***

Three cell dilutions mL^-1^ were tested; high (10^6^), low (10^3^), and lowest (10^2^) were tested and cell concentration was calculated through interpolation by standard curve. The expected cell concentration and calculated cell concentration were used to calculate the recovery percentage of the assay at different cell dilutions. Fig S8 shows the standard curve used for the calculation of cell concentrations. The following formulas were used for the recovery percentage and standard deviation of recovery percentage.

Recovery percentage = (calculated cell concentration)/ (Expected cell concentration) * 100

The recovery percentage at different cell dilutions:

Recovery percentage at 10^6^ cells = 85.8±15 %

Recovery percentage at 10^3^ cells = 98.2±9 %

Recovery percentage at 10^2^ cells = 96.8±10 %

Fig S8 Standard curve used for the calculation of recovery percentage to check the reproducibility of the assay.

***ST9 Recent studies for the dtection of pathogens in comparison with our study***

| **S.no.** | **Detection probes** | **Detection technique** | **Detection limit** | **Response time** | **Reference** |
| --- | --- | --- | --- | --- | --- |
| 1 | Paper based chromatography | Colorimetric | 10^4^cfu mL^-1^ | 12 hours | ^1^ |
| 2 | Aptamer and  carbon nano-tubes | Chemiluminescence | 10^3^cfu mL^-1^ | 16 hours | ^2^ |
| 3 | Antibody and Immunochromatographic strip | Enzyme linked-immunosorbant assay and Chromatography | 9.2 x 10^3^cfu mL^-1^ | 16 hours | ^3^ |
| 4 | Nanoparticle-enzyme | Electrochemical | 10^2^cfu mL^-1^ | 1 hour | ^4^ |
| 5 | Antibody-magnetic nanoparticle conjugate | Litmus test | 10^2^cfu mL^-1^ | 1 hours | ^5^ |
| 6 | Silver ions and antibody | pH indication and Electrochemical | 10^2^cfu mL^-1^ | 12 minutes | This work |

**References:**

1. Jokerst, J. C. *et al.* Development of a paper-based analytical device for colorimetric detection of select foodborne pathogens. *Anal. Chem.***84**, 2900–2907 (2012).

2. Yang, M. *et al.* Highly specific and cost-efficient detection of salmonella paratyphi A combining aptamers with single-walled carbon nanotubes. *Sensors (Switzerland)***13**, 6865–6881 (2013).

3. Park, S., Kim, Y. T. & Kim, Y. K. Optical enzyme-linked immunosorbent assay on a strip for detection of Salmonella typhimurium. *Biochip J.***4**, 110–116 (2010).

4. Chen, J. *et al.* Electrochemical nanoparticle-enzyme sensors for screening bacterial contamination in drinking water. *Analyst***140**, 4991–4996 (2015).

5. Chen, Q., Huang, F., Cai, G., Wang, M. & Lin, J. An optical biosensor using immunomagnetic separation, urease catalysis and pH indication for rapid and sensitive detection of Listeria monocytogenes. *Sensors Actuators, B Chem.***258**, 447–453 (2018).

6. Zaborska, W., Krajewska, B. & Olech, Z. Heavy metal ions inhibition of jack bean urease: Potential for rapid contaminant probing. *J. Enzyme Inhib. Med. Chem.***19**, 65–69 (2004).
